# Supplementary material for: Human vault RNAs exhibit diverse expression patterns and inter-locus compensation
Source: bioRxiv. 2026 Jul 18:2026.06.17.732918. Preprint. [Version 2] doi: 10.64898/2026.06.17.732918 (PMC13404743; doi:10.64898/2026.06.17.732918)
Supplement: Supplement 1 [file media-1.pdf]

Supplementary Information

Human vtRNAs exhibit diverse expression patterns and inter-locus compensation

Wayne O. Hemphill [1,2], *Arthur J. Zaug* [1,2], *Cody J. S. Hecht* [1,2], *Thomas R. Cech* [*,1,2]

[1] Department of Biochemistry, University of Colorado Boulder

[2] Howard Hughes Medical Institute and BioFrontiers Institute, University of Colorado Boulder

[*] Corresponding author: thomas.cech@colorado.edu

Synthetic Oligonucleotides Used

Name	*Sequence (5’ -> 3’)*
vt11	ACTCTCTTTTTGACTCACTCTTTGTGACG
	TCCTTTTACCATGGAAAGGGAGCTG
vt12	ACTGTTGTTTTGACTCACTTTGTGACG
	CTCTGTATTTCCTGCCCAGTGCTAAAATC
vt13	CACCGTTGTTTTGACTCACTCTTTGTG
	CTGCCCACTACTAAAATCAGCCATTTC
vt21	GGAGTTAGCTCAAGCGGTTACCT
	CCCAGCACAGAGATGGACAGATAG
wt	AAGGAATCAGGGCTCCTCGG
	GGGCCTCAGTAAAAAAGGGCG
ko	TGTCCCCAGATGGACAACTCCTC
	ACGAGGTGCTCCGTGTCC
adj	GCACAGACTGGCAAACTCAAAACAG
	ACCTGAAGTGCACATAATCCTGCC
CRISPR site 1a sgRNA	CACCTATAATCCGAAAGCAACCTC
	AAACGAGGTTGCTTTCGGATTATA
CRISPR site 1b sgRNA	CACCAGTCCTTTTACCTCTCTGGG
	AAACCCCAGAGAGGTAAAAGGACT
CRISPR site 2a sgRNA	CACCTATAATCCGGAGATAGCCTC
	AAACGAGGCTATCTCCGGATTATA
CRISPR site 2b sgRNA	CACCGAAGTTCAGCTCCTTTTCCA
	AAACTGGAAAAGGAGCTGAACTTC
CRISPR site 3a sgRNA	CACCTACAATCCGAAAGCAACCTC
	AAACGAGGTTGCTTTCGGATTGTA
CRISPR site 3b sgRNA	CACCAGCTCACTTTCCATAATAAA
	AAACTTTATTATGGAAAGTGAGCT
vtRNA1-1 probes	AGCGGTTACTTCGACAGTTCTTTAATTG
	AACAACCCAGACAGGTTGCTTG
vtRNA1-2 probes	AGCTCAGCGGTTACTTCGAGTAC
	CTGGAAAGCACCCGCGG
vtRNA1-3 probes	CAGCGGTTACTTCGCGTG
	ACAACCCAGAGAGGTGGTTTGATG
vtRNA2-1 probes	GCGAGACCGCATGACGC
	AGCCTCCATACTTTCTGTCACACC
SLFN11 probes	GTAGGGAAGTCCTGGGATGTGC
	GGGTTGGCAAAAATGAACACAAGG
GAPDH probes	GAGAAGGCTGGGGCTCATTT
	TGATGACCCTTTTGGCTCCC
GAPDH probes (Vir.)	GGAGCGAGATCCCTCCAAAAT
	GGCTGTTGTCATACTTCTCATGG
OC43 gRNA (RdRp) probes	GAGTGTAGATGCCCGTCTCG
	ATCAACACGCTGAAAACGGC
vtRNA1-1 template	AAAAGGACTGGAGAGCGCCCGCGGGTCTCGAACAACCCAGACAGGTTGCTTGTTTCAATTA<br>AAGAACTGTCGAAGTAACCGCTGAGCTAAAGCCAGCCTATAGTGAGTCGTATTA
vtRNA1-2 template	AAAAGAGCTGGAAAGCACCCGCGGGTCTCGAACCACCCAGAGAGGTGGTTACAATGTACT<br>CGAAGTAACCGCTGAGCTAAAGCCAGCCTATAGTGAGTCGTATTA
vtRNA1-3 template	AAGAGGGCTGGAGAGCGCCCGCGGGTCTCGAACAACCCAGAGAGGTGGTTTGATGACAC<br>GCGAAGTAACCGCTGAGCTAAAGCCAGCCTATAGTGAGTCGTATTA
vtRNA2-1 template	AAAAGGGTCAGTAAGCACCCGCGGGTCTCGAACCCCAGCACAGAGATGGACAGATAGAAA<br>GTCCGGCATGAGGAGGTAACCGCTTGAGCTAACTCCGACCTATAGTGAGTCGTATTA
T7 promoter	TAATACGACTCACTATA
VT11 insert	GATCTCTCACCAGTCAATAAAATATAATCCGAAAGCAACCTCCGGGCTGGCTTTAGCTCAGCGGTTACTT<br>CGACAGTTCTTTAATTGAAACAAGCAACCTGTCTGGGTTGTTCGAGACCCGCGGGCGCTCTCCAGTCCT<br>TTTACCTCTCTGGGCGGTTCGAGACCCGCGGGAGCTCTCCAGT
	AGCTACTGGAGAGCTCCCGCGGGTCTCGAACCGCCCAGAGAGGTAAAAGGACTGGAGAGCGCCCGCG<br>GGTCTCGAACAACCCAGACAGGTTGCTTGTTTCAATTAAAGAACTGTCGAAGTAACCGCTGAGCTAAAG<br>CCAGCCCGGAGGTTGCTTTCGGATTATATTTTATTGACTGGTGAGA

VT11del insert	GATCTCTCACCAGTCAATAAAATATAATCCGAAAGCTCCGGGCTGGCTTTAGCTCAGCGGTTACTTCGACAGTTCTTTAATTGAAACAAGCAACCTGTCTGGGTTGTTCGAGACCCGCGGGCGCTCTCCAGTCCTTTTACCTCTCTGGGCGGTTCGAGACCCGCGGGAGCTCTCCAGT
	AGCTACTGGAGAGCTCCCGCGGGTCTCGAACCGCCCAGAGAGGTAAAAGGACTGGAGAGCGCCCGCGGGTCTCGAACAACCCAGACAGGTTGCTTGTTTCAATTAAAGAACTGTCGAAGTAACCGCTGAGCTAAAGCCAGCCCGGAGCTTTCGGATTATATTTTATTGACTGGTGAGA
VT11 U6 insert	CACCGGCTGGCTTTAGCTCAGCGGTTACTTCGACAGTTCTTTAATTGAAACAAGCAACCTGTCTGGGTTGTTCGAGACCCGCGGGCGCTCTCCAGTCCTTTT
	AAAAAAAAGGACTGGAGAGCGCCCGCGGGTCTCGAACAACCCAGACAGGTTGCTTGTTTCAATTAAAGAACTGTCGAAGTAACCGCTGAGCTAAAGCCAGCC
VT21 insert	GATCCTCTTCTATGGTTTAGAAGTTTCAGTCGCACACTCCTACCCGGGTCGGAGTTAGCTCAAGCGGTTACCTCCTCATGCCGGACTTTCTATCTGTCCATCTCTGTGCTGGGGTTCGAGACCCGCGGGTGCTTACTGACCCTTTTATGCAATAAATTCGGTATAATCTGTCACTCTGAAGGCTTTGTTATT
	AGCTAATAACAAAGCCTTCAGAGTGACAGATTATACCGAATTTATTGCATAAAAGGGTCAGTAAGCACCCGCGGGTCTCGAACCCCAGCACAGAGATGGACAGATAGAAAGTCCGGCATGAGGAGGTAACCGCTTGAGCTAACTCCGACCCGGGTAGGAGTGTGCGACTGAAACTTCTAAACCATAGAAGAG

SUPPLEMENTAL TABLE 1. Synthetic oligos for these studies. A table of all synthetic oligos used in these studies, presented 5'→3' in Integrated DNA Technologies (IDT; Coralville, Iowa USA) sequence format. The second set of GAPDH probes ("Vir.") correspond to those used in OC43-associated RT-qPCR experiments.

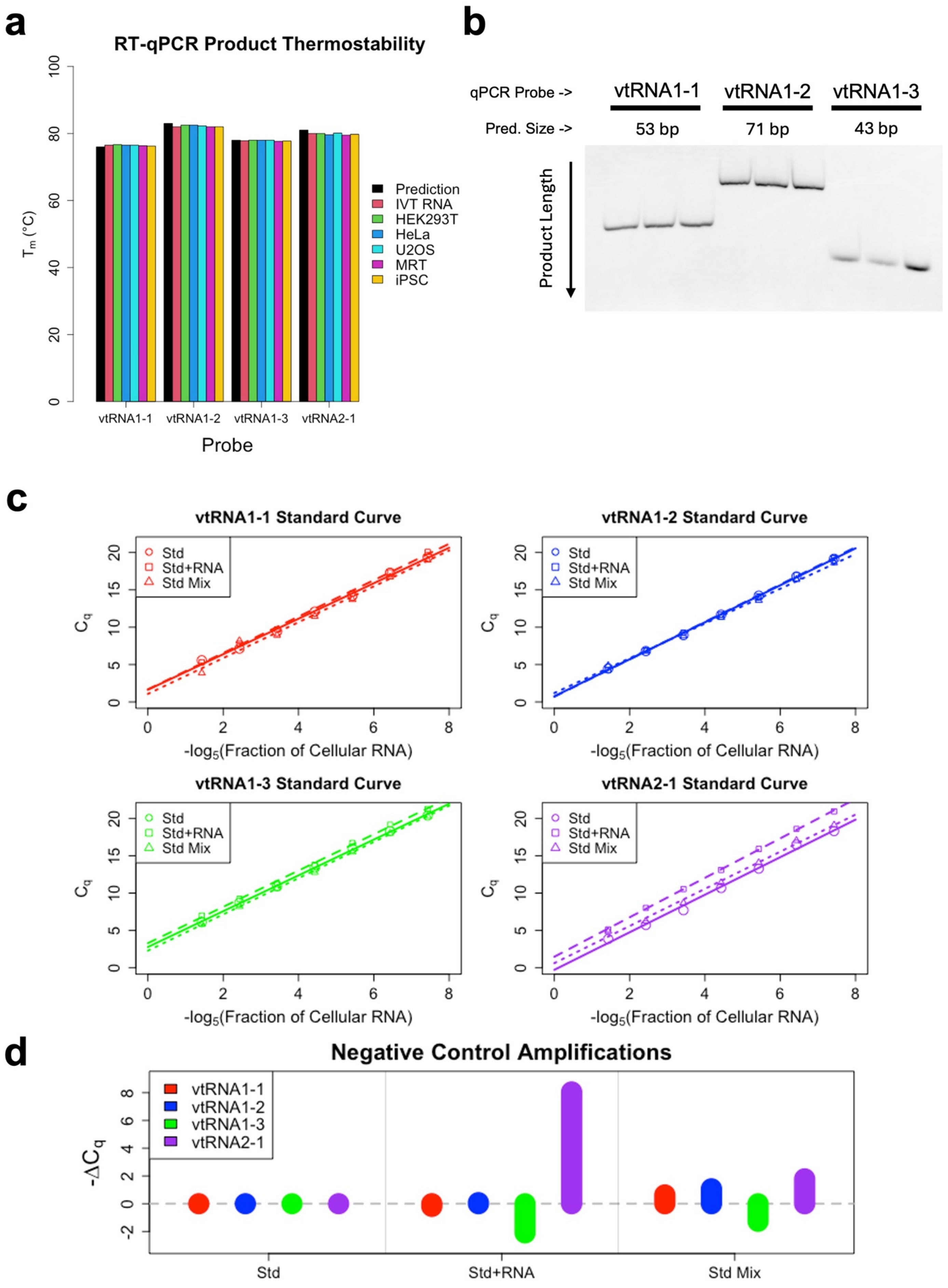
a
RT-qPCR Product Thermostability
Tm (°C)
Prediction
IVT RNA
HEK293T
HeLa
U2OS
MRT
iPSC
vtRNA1-1
vtRNA1-2
vtRNA1-3
vtRNA2-1
Probe
b
qPCR Probe ->
vtRNA1-1
vtRNA1-2
vtRNA1-3
Pred. Size ->
53 bp
71 bp
43 bp
Product Length
c
vtRNA1-1 Standard Curve
vtRNA1-2 Standard Curve
vtRNA1-3 Standard Curve
vtRNA2-1 Standard Curve
Std
Std+RNA
Std Mix
Cq
-log5(Fraction of Cellular RNA)
d
Negative Control Amplifications
-ΔCq
Std
Std+RNA
Std Mix

SUPPLEMENTAL FIGURE 1. RT-qPCR probes are highly specific for vtRNA1 paralogs. [a] Melt curves were generated for the amplification products from the Fig. 1a RT-qPCR experiment, and their melting temperatures (T_m) interpolated. “Prediction” refers to the theoretical T_m for the anticipated qPCR product, calculated via the GeneGlobe Tm predictor (Qiagen). “IVT RNA” refers to the T_m of samples from Fig. 1a standard curves. All other notation is consistent with Fig. 1a. Every data point is the average T_m of at least n=2 samples from Fig. 1a. **[b]** The HEK293T samples’ final qPCR amplification products were visualized on a polyacrylamide gel to compare their expected lengths (“Pred. Size”) to relative migration (“Product Length”), and to assess their homogeneity. **[c]** Standard curves were determined by subjecting the vtRNA paralogs’ IVT products were subjected to RT-qPCR alone (“Std”, solid lines), in the presence of total RNA from the vt1-TKO c2 cell line (“Std+RNA”, dashed lines), or in a 4-paralog mix of the standard curves (“Std Mix”, dotted lines), then fit with linear regression. **[d]** Relative C_q values of the zero-concentration samples in each vtRNA standard curve, normalized to the respective “Std” samples. The $-\Delta C_q$ values of the “Std+RNA” zero-concentration samples represent intrinsic vtRNA levels in the vt1-TKO c2 total RNA, while the “Std Mix” values are influenced by general variance.

VTRNA1-1 Gene Locus:

```
HEK293T_WT      ACTCTCTTTTTGACTCACTCTTTGTGACGTAGGTCTTTCTCACCAGTCAATAAAATATAA    60
CTRLdel         ACTCTCTTTTTGACTCACTCTTTGTGACGTAGGTCTTTCTCACCAGTCAATAAAATATAA    60
VTRNA1-1_KO     ACTCTCTTTTTGACTCACTCTTTGTGACGTAGGTCTTTCTCACCAGTCAATAAAATATAA    60
VTRNA1-2_KO     ACTCTCTTTTTGACTCACTCTTTGTGACGTAGGTCTTTCTCACCAGTCAATAAAATATAA    60
VTRNA1-3_KO     ACTCTCTTTTTGACTCACTCTTTGTGACGTAGGTCTTTCTCACCAGTCAATAAAATATAA    60
VTRNA1-TKOc1    ACTCTCTTTTTGACTCACTCTTTGTGACGTAGGTCTTTCTCACCAGTCAATAAAATATAA    60
VTRNA1-TKOc2    ACTCTCTTTTTGACTCACTCTTTGTGACGTAGGTCTTTCTCACCAGTCAATAAAATATAA    60
VTRNA1-TKOc3    ACTCTCTTTTTGACTCACTCTTTGTGACGTAGGTCTTTCTCACCAGTCAATAAAATATAA    60
HeLa            ACTCTCTTTTTGACTCACTCTTTGTGACGTAGGTCTTTCTCACCAGTCAATAAAATATAA    60
U2OS            ACTCTCTTTTTGACTCACTCTTTGTGACGTAGGTCTTTCTCACCAGTCAATAAAATATAA    60
MRT             ACTCTCTTTTTGACTCACTCTTTGTGACGTAGGTCTTTCTCACCAGTCAATAAAATATAA    60
BJ_Fib          ACTCTCTTTTTGACTCACTCTTTGTGACGTAGGTCTTTCTCACCAGTCAATAAAATATAA    60
iPSC            ACTCTCTTTTTGACTCACTCTTTGTGACGTAGGTCTTTCTCACCAGTCAATAAAATATAA    60

HEK293T_WT      TCCGAAAGCAACCTCCGGGCTGGCTTTAGCTCAGCGGTTACTTCGACAGTTCTTTAATTG    120
CTRLdel         TCCGAAAG----CTCCGGGCTGGCTTTAGCTCAGCGGTTACTTCGACAGTTCTTTAATTG    116
VTRNA1-1_KO     TCCGAAAGCAAC------------------------------------------------    72
VTRNA1-2_KO     TCCGAAAGCAACCTCCGGGCTGGCTTTAGCTCAGCGGTTACTTCGACAGTTCTTTAATTG    120
VTRNA1-3_KO     TCCGAAAGCAAC-TCCGGGCTGGCTTTAGCTCAGCGGTTACTTCGACAGTTCTTTAATTG    119
VTRNA1-TKOc1    TCCGAAAGCAAC------------------------------------------------    72
VTRNA1-TKOc2    TCCGAAAGCAAC------------------------------------------------    72
VTRNA1-TKOc3    TCCGAAAGCAAC------------------------------------------------    72
HeLa            TCCGAAAGCAACCTCCGGGCTGGCTTTAGCTCAGCGGTTACTTCGACAGTTCTTTAATTG    120
U2OS            TCCGAAAGCAACCTCCGGGCTGGCTTTAGCTCAGCGGTTACTTCGACAGTTCTTTAATTG    120
MRT             TCCGAAAGCAACCTCCGGGCTGGCTTTAGCTCAGCGGTTACTTCGACAGTTCTTTAATTG    120
BJ_Fib          TCCGAAAGCAACCTCCGGGCTGGCTTTAGCTCAGCGGTTACTTCGACAGTTCTTTAATTG    120
iPSC            TCCGAAAGCAACCTCCGGGCTGGCTTTAGCTCAGCGGTTACTTCGACAGTTCTTTAATTG    120

HEK293T_WT      AAACAAGCAACCTGTCTGGGTTGTTCGAGACCCGCGGGCGCTCTCCAGTCCTTTTACCTC    180
CTRLdel         AAACAAGCAACCTGTCTGGGTTGTTCGAGACCCGCGGGCGCTCTCCAGTCCTTTTACCTC    176
VTRNA1-1_KO     ------------------------------------------------------------    72
VTRNA1-2_KO     AAACAAGCAACCTGTCTGGGTTGTTCGAGACCCGCGGGCGCTCTCCAGTCCTTTTACCTC    180
```

```
VTRNA1-3_KO       AAACAAGCAACCTGTCTGGGTTGTTCGAGACCCGCGGGCGCTCTCCAGTCCTTTTACCTC     179
VTRNA1-TKOc1      ------------------------------------------------------------     72
VTRNA1-TKOc2      ------------------------------------------------------------     72
VTRNA1-TKOc3      ------------------------------------------------------------     72
HeLa              AAACAAGCAACCTGTCTGGGTTGTTCGAGACCCGCGGGCGCTCTCCAGTCCTTTTACCTC     180
U2OS              AAACAAGCAACCTGTCTGGGTTGTTCGAGACCCGCGGGCGCTCTCCAGTCCTTTTACCTC     180
MRT               AAACAAGCAACCTGTCTGGGTTGTTCGAGACCCGCGGGCGCTCTCCAGTCCTTTTACCTC     180
BJ_Fib            AAACAAGCAACCTGTCTGGGTTGTTCGAGACCCGCGGGCGCTCTCCAGTCCTTTTACCTC     180
iPSC              AAACAAGCAACCTGTCTGGGTTGTTCGAGACCCGCGGGCGCTCTCCAGTCCTTTTACCTC     180

HEK293T_WT        TCTGGGCGGTTCGAGACCCGCGGGAGCTCTCCAGTCCTTTTCACTGCTGAAGTTCAGCTC     240
CTRLdel           TCTGGGCGGTTCGAGACCCGCGGGAGCTCTCCAGTCCTTTTCACTGCTGAAGTTCAGCTC     236
VTRNA1-1_KO       --TGGGCGGTTCGAGACCCGCGGGAGCTCTCCAGTCCTTTTCACTGCTGAAGTTCAGCTC     130
VTRNA1-2_KO       TCTGGGCGGTTCGAGACCCGCGGGAGCTCTCCAGTCCTTTTCACTGCTGAAGTTCAGCTC     240
VTRNA1-3_KO       TCTGGGCGGTTCGAGACCCGCGGGAGCTCTCCAGTCCTTTTCACTGCTGAAGTTCAGCTC     239
VTRNA1-TKOc1      ------------------------------------------------------------     72
VTRNA1-TKOc2      ------------------------------------------------------------     72
VTRNA1-TKOc3      ------------------------------------------------------------     72
HeLa              TCTGGGCGGTTCGAGACCCGCGGGAGCTCTCCAGTCCTTTTCACTGCTGAAGTTCAGCTC     240
U2OS              TCTGGGCGGTTCGAGACCCGCGGGAGCTCTCCAGTCCTTTTCACTGCTGAAGTTCAGCTC     240
MRT               TCTGGGCGGTTCGAGACCCGCGGGAGCTCTCCAGTCCTTTTCACTGCTGAAGTTCAGCTC     240
BJ_Fib            TCTGGGCGGTTCGAGACCCGCGGGAGCTCTCCAGTCCTTTTCACTGCTGAAGTTCAGCTC     240
iPSC              TCTGGGCGGTTCGAGACCCGCGGGAGCTCTCCAGTCCTTTTCACTGCTGAAGTTCAGCTC     240

HEK293T_WT        CCTTTCCATGGTAAAAGGA     259
CTRLdel           CCTTTCCATGGTAAAAGGA     254
VTRNA1-1_KO       CCTTTCCATGGTAAAAGGA     148
VTRNA1-2_KO       CCTTTCCATGGTAAAAGGA     258
VTRNA1-3_KO       CCTTTCCATGGTAAAAGGA     257
VTRNA1-TKOc1      -------------------     71
VTRNA1-TKOc2      -------------------     71
VTRNA1-TKOc3      -------------------     71
HeLa              CCTTTCCATGGTAAAAGGA     259
U2OS              CCTTTCCATGGTAAAAGGA     259
```

```
MRT                 CCTTTCCATGGTAAAAGGA     259
BJ_Fib              CCTTTCCATGGTAAAAGGA     259
iPSC                CCTTTCCATGGTAAAAGGA     259
```

VTRNA1-2 Gene Locus:

```
HEK293T_WT          ACTGTTGTTTTGACTCACTTTGTGACGTAGGTCTTTCTCACCAGTCAAGAAAATATAATC      60
CTRLdel             ACTGTTGTTTTGACTCACTTTGTGACGTAGGTCTTTCTCACCAGTCAAGAAAATATAATC      60
VTRNA1-1_KO         ACTGTTGTTTTGACTCACTTTGTGACGTAGGTCTTTCTCACCAGTCAAGAAAATATAATC      60
VTRNA1-2_KO         ACTGTTGTTTTGACTCACTTTGTGACGTAGGTCTTTCTCACCAGTCAAGAAAATATAATC      60
VTRNA1-3_KO         ACTGTTGTTTTGACTCACTTTGTGACGTAGGTCTTTCTCACCAGTCAAGAAAATATAATC      60
VTRNA1-TKOc1        ------------------------------------------------------------      0
VTRNA1-TKOc2        ------------------------------------------------------------      0
VTRNA1-TKOc3        ------------------------------------------------------------      0
HeLa                ACTGTTGTTTTGACTCACTTTGTGACGTAGGTCTTTCTCACCAGTCAAGAAAATATAATC      60
U2OS                ACTGTTGTTTTGACTCACTTTGTGACGTAGGTCTTTCTCACCAGTCAAGAAAATATAATC      60
MRT                 ACTGTTGTTTTGACTCACTTTGTGACGTAGGTCTTTCTCACCAGTCAAGAAAATATAATC      60
BJ_Fib              ACTGTTGTTTTGACTCACTTTGTGACGTAGGTCTTTCTCACCAGTCAAGAAAATATAATC      60
iPSC                ACTGTTGTTTTGACTCACTTTGTGACGTAGGTCTTTCTCACCAGTCAAGAAAATATAATC      60

HEK293T_WT          CGGAGATAGCCTCTGGGCTGGCTTTAGCTCAGCGGTTACTTCGAGTACATTGTAACCACC      120
CTRLdel             CGGAGATAGCCTCTGGGCTGGCTTTAGCTCAGCGGTTACTTCGAGTACATTGTAACCACC      120
VTRNA1-1_KO         CGGAGATAGCCTCTGGGCTGGCTTTAGCTCAGCGGTTACTTCGAGTACATTGTAACCACC      120
VTRNA1-2_KO         CGGAGATAGC--------------------------------------------------      70
VTRNA1-3_KO         CGGAGATAGCCTCTGGGCTGGCTTTAGCTCAGCGGTTACTTCGAGTACATTGTAACCACC      120
VTRNA1-TKOc1        ------------------------------------------------------------      0
VTRNA1-TKOc2        ------------------------------------------------------------      0
VTRNA1-TKOc3        ------------------------------------------------------------      0
HeLa                CGGAGATAGCCTCTGGGCTGGCTTTAGCTCAGCGGTTACTTCGAGTACATTGTAACCACC      120
U2OS                CGGAGATAGCCTCTGGGCTGGCTTTAGCTCAGCGGTTACTTCGAGTACATTGTAACCACC      120
MRT                 CGGAGATAGCCTCTGGGCTGGCTTTAGCTCAGCGGTTACTTCGAGTACATTGTAACCACC      120
BJ_Fib              CGGAGATAGCCTCTGGGCTGGCTTTAGCTCAGCGGTTACTTCGAGTACATTGTAACCACC      120
iPSC                CGGAGATAGCCTCTGGGCTGGCTTTAGCTCAGCGGTTACTTCGAGTACATTGTAACCACC      120
```

```
HEK293T_WT       TCTCTGGGTGGTTCGAGACCCGCGGGTGCTTTCCAGCTCTTTTACTGCTGAAGTTCAGCT      180
CTRLdel          TCTCTGGGTGGTTCGAGACCCGCGGGTGCTTTCCAGCTCTTTTACTGCTGAAGTTCAGCT      180
VTRNA1-1_KO      TCTCTGGGTGGTTCGAGACCCGCGGGTGCTTTCCAGCTCTTTTACTGCTGAAGTTCAGCT      180
VTRNA1-2_KO      ------------------------------------------------------------      70
VTRNA1-3_KO      TCTCTGGGTGGTTCGAGACCCGCGGGTGCTTTCCAGCTCTTTTACTGCTGAAGTTCAGCT      180
VTRNA1-TKOc1     ------------------------------------------------------------      0
VTRNA1-TKOc2     ------------------------------------------------------------      0
VTRNA1-TKOc3     ------------------------------------------------------------      0
HeLa             TCTCTGGGTGGTTCGAGACCCGCGGGTGCTTTCCAGCTCTTTTACTGCTGAAGTTCAGCT      180
U2OS             TCTCTGGGTGGTTCGAGACCCGCGGGTGCTTTCCAGCTCTTTTACTGCTGAAGTTCAGCT      180
MRT              TCTCTGGGTGGTTCGAGACCCGCGGGTGCTTTCCAGCTCTTTTACTGCTGAAGTTCAGCT      180
BJ_Fib           TCTCTGGGTGGTTCGAGACCCGCGGGTGCTTTCCAGCTCTTTTACTGCTGAAGTTCAGCT      180
iPSC             TCTCTGGGTGGTTCGAGACCCGCGGGTGCTTTCCAGCTCTTTTACTGCTGAAGTTCAGCT      180

HEK293T_WT       CCTTTTCCATGGGGAACCATGGAGAAATGGCTGATTTTAGCACTGGGCAGGAAATACAGA      240
CTRLdel          CCTTTTCCATGGGGAACCATGGAGAAATGGCTGATTTTAGCACTGGGCAGGAAATACAGA      240
VTRNA1-1_KO      CCTTTTCCATGGGGAACCATGGAGAAATGGCTGATTTTAGCACTGGGCAGGAAATACAGA      240
VTRNA1-2_KO      -----TCCATGGGGAACCATGGAGAAATGGCTGATTTTAGCACTGGGCAGGAAATACAGA      125
VTRNA1-3_KO      CCTTTTCCATGGGGAACCATGGAGAAATGGCTGATTTTAGCACTGGGCAGGAAATACAGA      240
VTRNA1-TKOc1     ------------------------------------------------------------      0
VTRNA1-TKOc2     ------------------------------------------------------------      0
VTRNA1-TKOc3     ------------------------------------------------------------      0
HeLa             CCTTTTCCATGGGGAACCATGGAGAAATGGCTGATTTTAGCACTGGGCAGGAAATACAGA      240
U2OS             CCTTTTCCATGGGGAACCATGGAGAAATGGCTGATTTTAGCACTGGGCAGGAAATACAGA      240
MRT              CCTTTTCCATGGGGAACCATGGAGAAATGGCTGATTTTAGCACTGGGCAGGAAATACAGA      240
BJ_Fib           CCTTTTCCATGGGGAACCATGGAGAAATGGCTGATTTTAGCACTGGGCAGGAAATACAGA      240
iPSC             CCTTTTCCATGGGGAACCATGGAGAAATGGCTGATTTTAGCACTGGGCAGGAAATACAGA      240

HEK293T_WT       G      241
CTRLdel          G      241
VTRNA1-1_KO      G      241
VTRNA1-2_KO      G      126
VTRNA1-3_KO      G      241
```

```
VTRNA1-TKOc1     -     0
VTRNA1-TKOc2     -     0
VTRNA1-TKOc3     -     0
HeLa             G     241
U2OS             G     241
MRT              G     241
BJ_Fib           G     241
iPSC             G     241
```

VTRNA1-3 Gene Locus:

```
HEK293T_WT       CACCGTTGTTTTGACTCACTCTTTGTGACGTAGGTCTTTCTCACCAGTCAAGAAAATACA      60
CTRLdel          CACCGTTGTTTTGACTCACTCTTTGTGACGTAGGTCTTTCTCACCAGTCAAGAAAATACA      60
VTRNA1-1_KO      CACCGTTGTTTTGACTCACTCTTTGTGACGTAGGTCTTTCTCACCAGTCAAGAAAATACA      60
VTRNA1-2_KO      CACCGTTGTTTTGACTCACTCTTTGTGACGTAGGTCTTTCTCACCAGTCAAGAAAATACA      60
VTRNA1-3_KO      CACCGTTGTTTTGACTCACTCTTTGTGACGTAGGTCTTTCTCACCAGTCAAGAAAATACA      60
VTRNA1-TKOc1     ------------------------------------------------------------      0
VTRNA1-TKOc2     ------------------------------------------------------------      0
VTRNA1-TKOc3     ------------------------------------------------------------      0
HeLa             CACCGTTGTTTTGACTCACTCTTTGTGACGTAGGTCTTTCTCACCAGTCAAGAAAATACA      60
U2OS             CACCGTTGTTTTGACTCACTCTTTGTGACGTAGGTCTTTCTCACCAGTCAAGAAAATACA      60
MRT              CACCGTTGTTTTGACTCACTCTTTGTGACGTAGGTCTTTCTCACCAGTCAAGAAAATACA      60
BJ_Fib           CACCGTTGTTTTGACTCACTCTTTGTGACGTAGGTCTTTCTCACCAGTCAAGAAAATACA      60
iPSC             CACCGTTGTTTTGACTCACTCTTTGTGACGTAGGTCTTTCTCACCAGTCAAGAAAATACA      60

HEK293T_WT       ATCCGAAAGCAACCTCTGGGCTGGCTTTAGCTCAGCGGTTACTTCGCGTGTCATCAAACC      120
CTRLdel          ATCCGAAAG----CTCTGGGCTGGCTTTAGCTCAGCGGTTACTTCGCGTGTCATCAAACC      116
VTRNA1-1_KO      ATCCGAAAGCAACCTCTGGGCTGGCTTTAGCTCAGCGGTTACTTCGCGTGTCATCAAACC      120
VTRNA1-2_KO      ATCCGAAAGCAACCTCTGGGCTGGCTTTAGCTCAGCGGTTACTTCGCGTGTCATCAAACC      120
VTRNA1-3_KO      ATCCGAAAGCAAC-----------------------------------------------      73
VTRNA1-TKOc1     ------------------------------------------------------------      0
VTRNA1-TKOc2     ------------------------------------------------------------      0
VTRNA1-TKOc3     ------------------------------------------------------------      0
```

HeLa	ATCCGAAAGCAACCTCTGGGCTGGCTTTAGCTCAGCGGTTACTTCGCGTGTCATCAAACC	120
U2OS	ATCCGAAAGCAACCTCTGGGCTGGCTTTAGCTCAGCGGTTACTTCGCGTGTCATCAAACC	120
MRT	ATCCGAAAGCAACCTCTGGGCTGGCTTTAGCTCAGCGGTTACTTCGCGTGTCATCAAACC	120
BJ_Fib	ATCCGAAAGCAACCTCTGGGCTGGCTTTAGCTCAGCGGTTACTTCGCGTGTCATCAAACC	120
iPSC	ATCCGAAAGCAACCTCTGGGCTGGCTTTAGCTCAGCGGTTACTTCGCGTGTCATCAAACC	120

HEK293T_WT	ACCTCTCTGGGTTGTTCGAGACCCGCGGGCGCTCTCCAGCCCTCTTACTGCTGAAGTTCA	180
CTRLdel	ACCTCTCTGGGTTGTTCGAGACCCGCGGGCGCTCTCCAGCCCTCTTACTGCTGAAGTTCA	176
VTRNA1-1_KO	ACCTCTCTGGGTTGTTCGAGACCCGCGGGCGCTCTCCAGCCCTCTTACTGCTGAAGTTCA	180
VTRNA1-2_KO	ACCTCTCTGGGTTGTTCGAGACCCGCGGGCGCTCTCCAGCCCTCTTACTGCTGAAGTTCA	180
VTRNA1-3_KO	--	73
VTRNA1-TKOc1	--	0
VTRNA1-TKOc2	--	0
VTRNA1-TKOc3	--	0
HeLa	ACCTCTCTGGGTTGTTCGAGACCCGCGGGCGCTCTCCAGCCCTCTTACTGCTGAAGTTCA	180
U2OS	ACCTCTCTGGGTTGTTCGAGACCCGCGGGCGCTCTCCAGCCCTCTTACTGCTGAAGTTCA	180
MRT	ACCTCTCTGGGTTGTTCGAGACCCGCGGGCGCTCTCCAGCCCTCTTACTGCTGAAGTTCA	180
BJ_Fib	ACCTCTCTGGGTTGTTCGAGACCCGCGGGCGCTCTCCAGCCCTCTTACTGCTGAAGTTCA	180
iPSC	ACCTCTCTGGGTTGTTCGAGACCCGCGGGCGCTCTCCAGCCCTCTTACTGCTGAAGTTCA	180

HEK293T_WT	GCTCACTTTCCATAATAAAAGGATCCAGGGTTTCTTGGAGAAATGGCTGATTTTAGTAGT	240
CTRLdel	GCTCACTTTCCAT--TAAAAGGATCCAGGGTTTCTTGGAGAAATGGCTGATTTTAGTAGT	234
VTRNA1-1_KO	GCTCACTTTCCATAATAAAAGGATCCAGGGTTTCTTGGAGAAATGGCTGATTTTAGTAGT	240
VTRNA1-2_KO	GCTCACTTTCCATAATAAAAGGATCCAGGGTTTCTTGGAGAAATGGCTGATTTTAGTAGT	240
VTRNA1-3_KO	---------------TAAAAGGATCCAGGGTTTCTTGGAGAAATGGCTGATTTTAGTAGT	118
VTRNA1-TKOc1	----------------AAAAGGATCCAGGGTTTCTTGGAGAAATGGCTGATTTTAGTAGT	44
VTRNA1-TKOc2	----------------AAAAGGATCCAGGGTTTCTTGGAGAAATGGCTGATTTTAGTAGT	44
VTRNA1-TKOc3	---------------TAAAAGGATCCAGGGTTTCTTGGAGAAATGGCTGATTTTAGTAGT	45
HeLa	GCTCACTTTCCATAATAAAAGGATCCAGGGTTTCTTGGAGAAATGGCTGATTTTAGTAGT	240
U2OS	GCTCACTTTCCATAATAAAAGGATCCAGGGTTTCTTGGAGAAATGGCTGATTTTAGTAGT	240
MRT	GCTCACTTTCCATAATAAAAGGATCCAGGGTTTCTTGGAGAAATGGCTGATTTTAGTAGT	240
BJ_Fib	GCTCACTTTCCATAATAAAAGGATCCAGGGTTTCTTGGAGAAATGGCTGATTTTAGTAGT	240
iPSC	GCTCACTTTCCATAATAAAAGGATCCAGGGTTTCTTGGAGAAATGGCTGATTTTAGTAGT	240

```
HEK293T_WT        GGGCAG    246
CTRLdel           GGGCAG    240
VTRNA1-1_KO       GGGCAG    246
VTRNA1-2_KO       GGGCAG    246
VTRNA1-3_KO       GGGCAG    124
VTRNA1-TKOc1      GGGCAG    50
VTRNA1-TKOc2      GGGCAG    50
VTRNA1-TKOc3      GGGCAG    51
HeLa              GGGCAG    246
U2OS              GGGCAG    246
MRT               GGGCAG    246
BJ_Fib            GGGCAG    246
iPSC              GGGCAG    246
```

VTRNA2-1 Gene Locus:

```
HEK293T_WT        GCGAGACCGCATGACGCAGGCCTCTCGGGGGCGGGGAGCACATGCAGCCCGTCCCTCTCC    60
CTRLdel           GCGAGACCGCATGACGCAGGCCTCTCGGGGGCGGGGAGCACATGCAGCCCGTCCCTCTCC    60
VTRNA1-1_KO       GCGAGACCGCATGACGCAGGCCTCTCGGGGGCGGGGAGCACATGCAGCCCGTCCCTCTCC    60
VTRNA1-2_KO       GCGAGACCGCATGACGCAGGCCTCTCGGGGGCGGGGAGCACATGCAGCCCGTCCCTCTCC    60
VTRNA1-3_KO       GCGAGACCGCATGACGCAGGCCTCTCGGGGGCGGGGAGCACATGCAGCCCGTCCCTCTCC    60
VTRNA1-TKOc1      GCGAGACCGCATGACGCAGGCCTCTCGGGGGCGGGGAGCACATGCAGCCCGTCCCTCTCC    60
VTRNA1-TKOc2      GCGAGACCGCATGACGCAGGCCTCTCGGGGGCGGGGAGCACATGCAGCCCGTCCCTCTCC    60
VTRNA1-TKOc3      GCGAGACCGCATGACGCAGGCCTCTCGGGGGCGGGGAGCACATGCAGCCCGTCCCTCTCC    60
HeLa              GCGAGACCGCATGACGCAGGCCTCTCGGGGGCGGGGAGCACATGCAGCCCGTCCCTCTCC    60
U2OS              GCGAGACCGCATGACGCAGGCCTCTCGGGGGCGGGGAGCACATGCAGCCCGTCCCTCTCC    60
MRT               GCGAGACCGCATGACGCAGGCCTCTCGGGGGCGGGGAGCACATGCAGCCCGTCCCTCTCC    60
BJ_Fib            GCGAGACCGCATGACGCAGGCCTCTCGGGGGCGGGGAGCACATGCAGCCCGTCCCTCTCC    60
iPSC              GCGAGACCGCATGACGCAGGCCTCTCGGGGGCGGGGAGCACATGCAGCCCGTCCCTCTCC    60

HEK293T_WT        ACATCGTCACTCTTCTATGGTTTAGAAGTTTCAGTCGCACACTCCTACCCGGGTCGGAGT    120
CTRLdel           ACATCGTCACTCTTCTATGGTTTAGAAGTTTCAGTCGCACACTCCTACCCGGGTCGGAGT    120
VTRNA1-1_KO       ACATCGTCACTCTTCTATGGTTTAGAAGTTTCAGTCGCACACTCCTACCCGGGTCGGAGT    120
```

```
VTRNA1-2_KO      ACATCGTCACTCTTCTATGGTTTAGAAGTTTCAGTCGCACACTCCTACCCGGGTCGGAGT    120
VTRNA1-3_KO      ACATCGTCACTCTTCTATGGTTTAGAAGTTTCAGTCGCACACTCCTACCCGGGTCGGAGT    120
VTRNA1-TKOc1     ACATCGTCACTCTTCTATGGTTTAGAAGTTTCAGTCGCACACTCCTACCCGGGTCGGAGT    120
VTRNA1-TKOc2     ACATCGTCACTCTTCTATGGTTTAGAAGTTTCAGTCGCACACTCCTACCCGGGTCGGAGT    120
VTRNA1-TKOc3     ACATCGTCACTCTTCTATGGTTTAGAAGTTTCAGTCGCACACTCCTACCCGGGTCGGAGT    120
HeLa             ACATCGTCACTCTTCTATGGTTTAGAAGTTTCAGTCGCACACTCCTACCCGGGTCGGAGT    120
U2OS             ACATCGTCACTCTTCTATGGTTTAGAAGTTTCAGTCGCACACTCCTACCCGGGTCGGAGT    120
MRT              ACATCGTCACTCTTCTATGGTTTAGAAGTTTCAGTCGCACACTCCTACCCGGGTCGGAGT    120
BJ_Fib           ACATCGTCACTCTTCTATGGTTTAGAAGTTTCAGTCGCACACTCCTACCCGGGTCGGAGT    120
iPSC             ACATCGTCACTCTTCTATGGTTTAGAAGTTTCAGTCGCACACTCCTACCCGGGTCGGAGT    120

HEK293T_WT       TAGCTCAAGCGGTTACCTCCTCATGCCGGACTTTCTATCTGTCCATCTCTGTGCTGGGGT    180
CTRLdel          TAGCTCAAGCGGTTACCTCCTCATGCCGGACTTTCTATCTGTCCATCTCTGTGCTGGGGT    180
VTRNA1-1_KO      TAGCTCAAGCGGTTACCTCCTCATGCCGGACTTTCTATCTGTCCATCTCTGTGCTGGGGT    180
VTRNA1-2_KO      TAGCTCAAGCGGTTACCTCCTCATGCCGGACTTTCTATCTGTCCATCTCTGTGCTGGGGT    180
VTRNA1-3_KO      TAGCTCAAGCGGTTACCTCCTCATGCCGGACTTTCTATCTGTCCATCTCTGTGCTGGGGT    180
VTRNA1-TKOc1     TAGCTCAAGCGGTTACCTCCTCATGCCGGACTTTCTATCTGTCCATCTCTGTGCTGGGGT    180
VTRNA1-TKOc2     TAGCTCAAGCGGTTACCTCCTCATGCCGGACTTTCTATCTGTCCATCTCTGTGCTGGGGT    180
VTRNA1-TKOc3     TAGCTCAAGCGGTTACCTCCTCATGCCGGACTTTCTATCTGTCCATCTCTGTGCTGGGGT    180
HeLa             TAGCTCAAGCGGTTACCTCCTCATGCCGGACTTTCTATCTGTCCATCTCTGTGCTGGGGT    180
U2OS             TAGCTCAAGCGGTTACCTCCTCATGCCGGACTTTCTATCTGTCCATCTCTGTGCTGGGGT    180
MRT              TAGCTCAAGCGGTTACCTCCTCATGCCGGACTTTCTATCTGTCCATCTCTGTGCTGGGGT    180
BJ_Fib           TAGCTCAAGCGGTTACCTCCTCATGCCGGACTTTCTATCTGTCCATCTCTGTGCTGGGGT    180
iPSC             TAGCTCAAGCGGTTACCTCCTCATGCCGGACTTTCTATCTGTCCATCTCTGTGCTGGGGT    180

HEK293T_WT       TCGAGACCCGCGGGTGCTTACTGACCCTTTTATGCAATAAATTCGGTATAATCTGTCACT    240
CTRLdel          TCGAGACCCGCGGGTGCTTACTGACCCTTTTATGCAATAAATTCGGTATAATCTGTCACT    240
VTRNA1-1_KO      TCGAGACCCGCGGGTGCTTACTGACCCTTTTATGCAATAAATTCGGTATAATCTGTCACT    240
VTRNA1-2_KO      TCGAGACCCGCGGGTGCTTACTGACCCTTTTATGCAATAAATTCGGTATAATCTGTCACT    240
VTRNA1-3_KO      TCGAGACCCGCGGGTGCTTACTGACCCTTTTATGCAATAAATTCGGTATAATCTGTCACT    240
VTRNA1-TKOc1     TCGAGACCCGCGGGTGCTTACTGACCCTTTTATGCAATAAATTCGGTATAATCTGTCACT    240
VTRNA1-TKOc2     TCGAGACCCGCGGGTGCTTACTGACCCTTTTATGCAATAAATTCGGTATAATCTGTCACT    240
VTRNA1-TKOc3     TCGAGACCCGCGGGTGCTTACTGACCCTTTTATGCAATAAATTCGGTATAATCTGTCACT    240
HeLa             TCGAGACCCGCGGGTGCTTACTGACCCTTTTATGCAATAAATTCGGTATAATCTGTCACT    240
```

```
U2OS            TCGAGACCCGCGGGTGCTTACTGACCCTTTTATGCAATAAATTCGGTATAATCTGTCACT      240
MRT             TCGAGACCCGCGGGTGCTTACTGACCCTTTTATGCAATAAATTCGGTATAATCTGTCACT      240
BJ_Fib          TCGAGACCCGCGGGTGCTTACTGACCCTTTTATGCAATAAATTCGGTATAATCTGTCACT      240
iPSC            TCGAGACCCGCGGGTGCTTACTGACCCTTTTATGCAATAAATTCGGTATAATCTGTCACT      240

HEK293T_WT      CTGAAGGCTTTGTTATTTTTTATCCCTTTTAACTTTGCTAAATTAATAGGCTGATAACAT      300
CTRLdel         CTGAAGGCTTTGTTATTTTTTATCCCTTTTAACTTTGCTAAATTAATAGGCTGATAACAT      300
VTRNA1-1_KO     CTGAAGGCTTTGTTATTTTTTATCCCTTTTAACTTTGCTAAATTAATAGGCTGATAACAT      300
VTRNA1-2_KO     CTGAAGGCTTTGTTATTTTTTATCCCTTTTAACTTTGCTAAATTAATAGGCTGATAACAT      300
VTRNA1-3_KO     CTGAAGGCTTTGTTATTTTTTATCCCTTTTAACTTTGCTAAATTAATAGGCTGATAACAT      300
VTRNA1-TKOc1    CTGAAGGCTTTGTTATTTTTTATCCCTTTTAACTTTGCTAAATTAATAGGCTGATAACAT      300
VTRNA1-TKOc2    CTGAAGGCTTTGTTATTTTTTATCCCTTTTAACTTTGCTAAATTAATAGGCTGATAACAT      300
VTRNA1-TKOc3    CTGAAGGCTTTGTTATTTTTTATCCCTTTTAACTTTGCTAAATTAATAGGCTGATAACAT      300
HeLa            CTGAAGGCTTTGTTATTTTTTATCCCTTTTAACTTTGCTAAATTAATAGGCTGATAACAT      300
U2OS            CTGAAGGCTTTGTTATTTTTTATCCCTTTTAACTTTGCTAAATTAATAGGCTGATAACAT      300
MRT             CTGAAGGCTTTGTTATTTTTTATCCCTTTTAACTTTGCTAAATTAATAGGCTGATAACAT      300
BJ_Fib          CTGAAGGCTTTGTTATTTTTTATCCCTTTTAACTTTGCTAAATTAATAGGCTGATAACAT      300
iPSC            CTGAAGGCTTTGTTATTTTTTATCCCTTTTAACTTTGCTAAATTAATAGGCTGATAACAT      300

HEK293T_WT      AAGTTGGTGTCACTTTGAAGGTGTGACAGAAAGTATGGAGGCT      343
CTRLdel         AAGTTGGTGTCACTTTGAAGGTGTGACAGAAAGTATGGAGGCT      343
VTRNA1-1_KO     AAGTTGGTGTCACTTTGAAGGTGTGACAGAAAGTATGGAGGCT      343
VTRNA1-2_KO     AAGTTGGTGTCACTTTGAAGGTGTGACAGAAAGTATGGAGGCT      343
VTRNA1-3_KO     AAGTTGGTGTCACTTTGAAGGTGTGACAGAAAGTATGGAGGCT      343
VTRNA1-TKOc1    AAGTTGGTGTCACTTTGAAGGTGTGACAGAAAGTATGGAGGCT      343
VTRNA1-TKOc2    AAGTTGGTGTCACTTTGAAGGTGTGACAGAAAGTATGGAGGCT      343
VTRNA1-TKOc3    AAGTTGGTGTCACTTTGAAGGTGTGACAGAAAGTATGGAGGCT      343
HeLa            AAGTTGGTGTCACTTTGAAGGTGTGACAGAAAGTATGGAGGCT      343
U2OS            AAGTTGGTGTCACTTTGAAGGTGTGACAGAAAGTATGGAGGCT      343
MRT             AAGTTGGTGTCACTTTGAAGGTGTGACAGAAAGTATGGAGGCT      343
BJ_Fib          AAGTTGGTGTCACTTTGAAGGTGTGACAGAAAGTATGGAGGCT      343
iPSC            AAGTTGGTGTCACTTTGAAGGTGTGACAGAAAGTATGGAGGCT      343
```

SUPPLEMENTAL FIGURE 2. Alignment of Nanopore sequencing results for VTRNA1 and 2 loci of various human cell lines. Wild-type, CTRLdel, and vtRNA single-knockout cell lines were subjected to PCR with the vt11, vt12, vt13, and vt21 primer sets (Fig. 2a), and vtRNA triple-knockout cell lines were subjected to PCR with the ko and vt21 primer sets (Fig. 2a). Products were Nanopore sequenced and dominant sequences were aligned across the four vtRNA paralogs' genetic loci with the Clustal Omega Multiple Sequence Alignment (EMBL-EBI). Cas9 cleavage sites are highlighted in purple, vtRNA coding strand sequences in green, and transcription start and termination sites in blue and red, respectively.

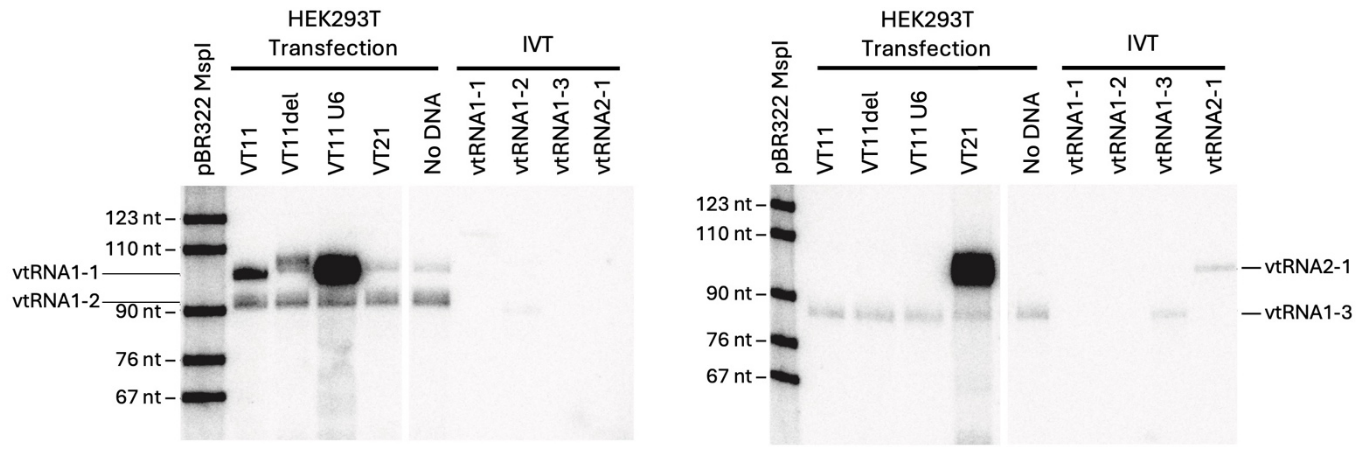

SUPPLEMENTAL FIGURE 3. vtRNA2-1 expression in HEK293T cells is suppressed epigenetically. Northern blots probing for vtRNA1-1 and 1-2 (left), and vtRNA1-3 and vtRNA2-1 (right) in cellular RNA extracted from wild-type HEK293T cells transfected with vtRNA-expressing plasmids. Constructs expressed vtRNAs using their natural, internal promoters (pUC19 vector + gene with 40 bp flanking sequences); that is, VT11 is N_{40}[VTRNA1-1]N_{40}, VT11del is $N_{31}\Delta_4N_5$[VTRNA1-1]N_{40}, and VT21 is N_{40}[VTRNA2-1]N_{40}. A no-transfection control (No DNA) and a positive control with *VTRNA1-1* behind a synthetic U6 promoter (VT11 U6) are included. The negative (no-transfection) control shows the basal levels of vtRNAs in the wild-type cells. “HEK293T Transfection” lanes are loaded with equal amounts of cellular RNA. IVT RNA standards (“IVT”) are included, and the anticipated vtRNA product lengths are indicated by labels to the left/right of the gels. White, dividing lines in gels are from trimming extraneous lanes; images are cropped portions of the same parent image, without alterations to contrast, alignment, etc. Data are representative of three independent experiments.

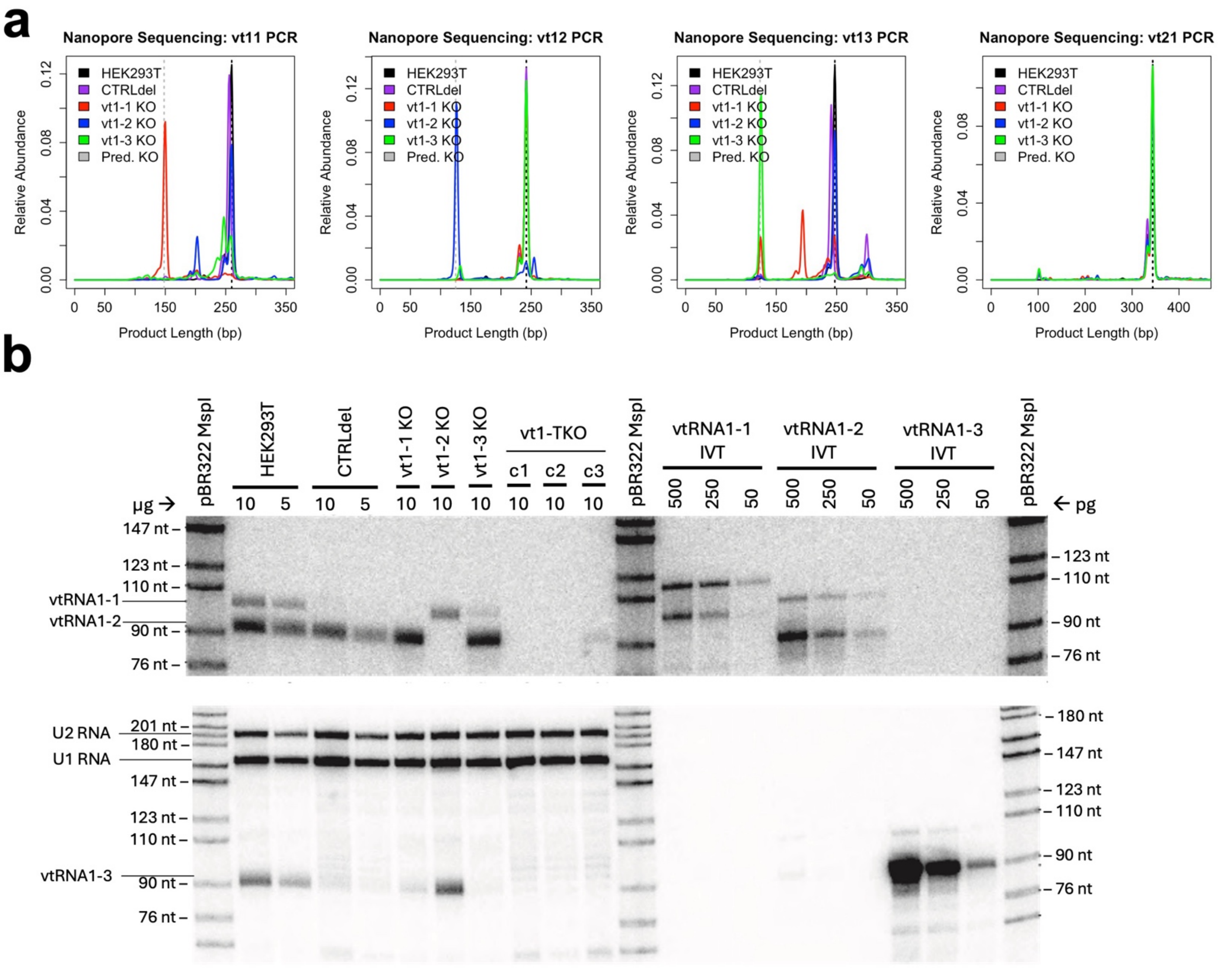

SUPPLEMENTAL FIGURE 4. Validation of vtRNA1-1, 1-2, 1-3, and 1-TKO HEK293T cell lines. [a] Product size distributions from Nanopore sequencing of Fig. 2b PCR reactions. Predicted product lengths for knockout cell lines ("Pred. KO"; vertical, grey dashed lines) are based on the Cas9 cleavage sites shown in Supplemental Figure 2. Wild-type length products (vertical, black dashed lines) are based on the dominant band in HEK293T cell line. [b] Northern blots probing for vtRNA1-1 and 1-2 (top), and vtRNA1-3 (bottom) in wild-type, CTRLdel, and vtRNA knockout HEK293T cell lines, and in IVT RNA standards ("IVT").

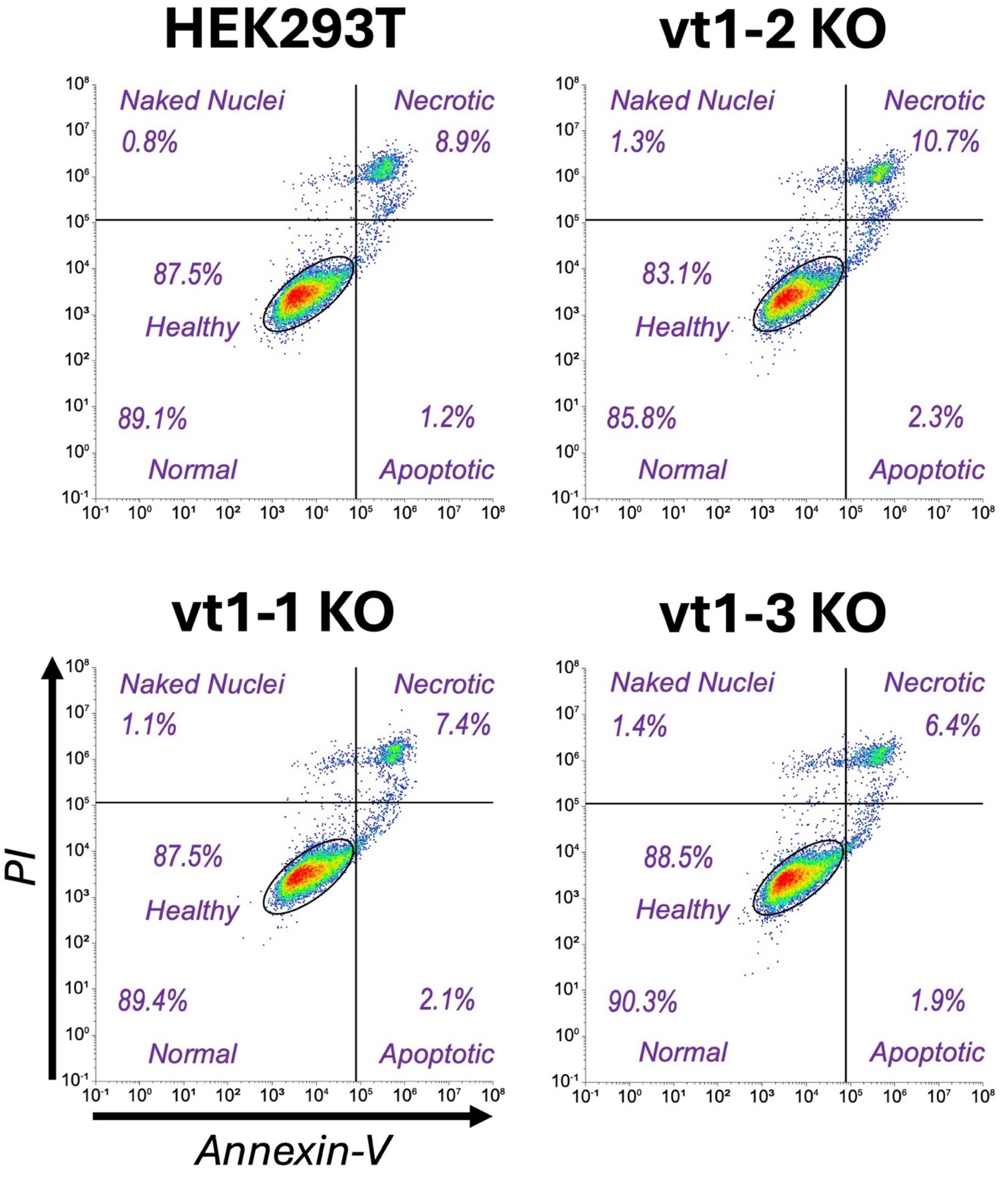

SUPPLEMENTAL FIGURE 5. Cell viability of HEK293T lacking vtRNA1 paralogs is not compromised. Wild-type or vtRNA1 single-knockout HEK293T cells were grown under standard conditions, dual stained with propidium iodide (PI) and Annexin-V, then analyzed via flow cytometry. Events shown were first gated by SSC-A/FSC-A then FSC-A/FSC-H to identify singlets. Gating and graphics were via the Floreada web server.

a

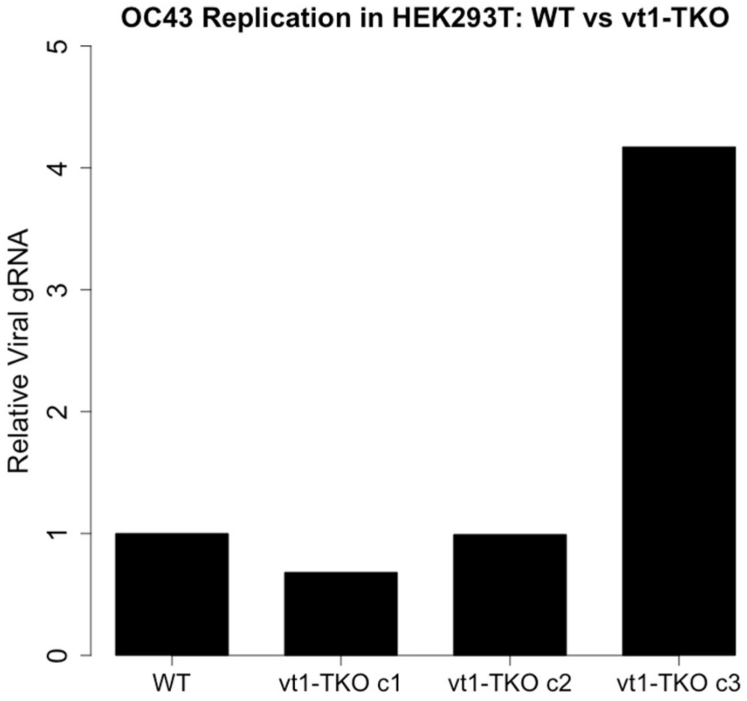
OC43 Replication in HEK293T: WT vs vt1-TKO
Relative Viral gRNA
0
1
2
3
4
5
WT
vt1-TKO c1
vt1-TKO c2
vt1-TKO c3

b

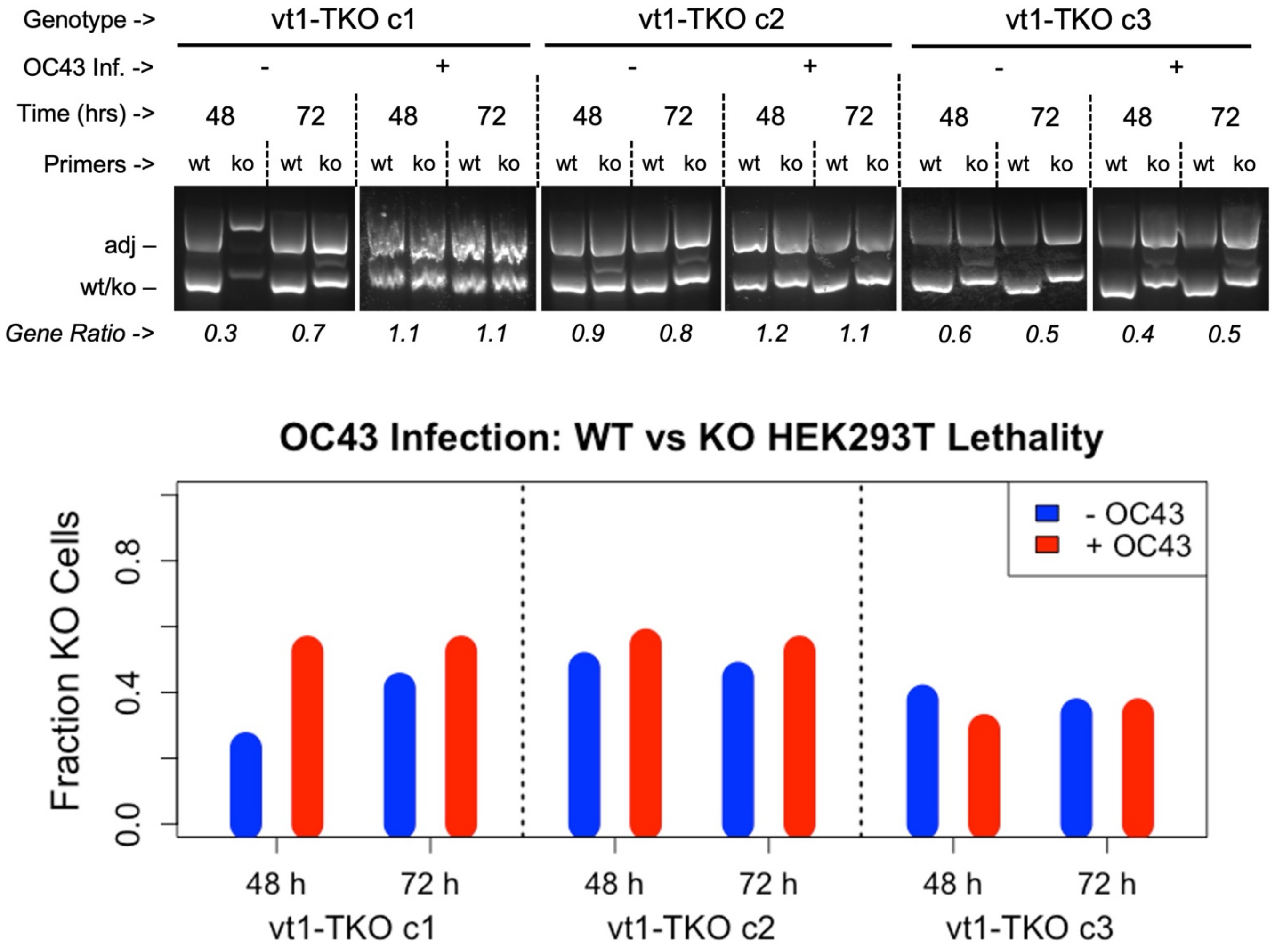
Genotype ->
vt1-TKO c1
vt1-TKO c2
vt1-TKO c3
OC43 Inf. ->
- + - + - +
Time (hrs) ->
48 72 48 72 48 72 48 72 48 72 48 72
Primers ->
wt ko wt ko wt ko wt ko wt ko wt ko wt ko wt ko wt ko wt ko wt ko wt ko
adj –
wt/ko –
Gene Ratio ->
0.3 0.7 1.1 1.1 0.9 0.8 1.2 1.1 0.6 0.5 0.4 0.5
OC43 Infection: WT vs KO HEK293T Lethality
Fraction KO Cells
0.0
0.4
0.8
- OC43
+ OC43
48 h
72 h
48 h
72 h
48 h
72 h
vt1-TKO c1
vt1-TKO c2
vt1-TKO c3

SUPPLEMENTAL FIGURE 6. Loss of vtRNA1 paralogs does not affect OC43 coronavirus replication or survival of HEK293T cells. [a] Wild-type (WT) and vt1-TKO HEK293T cell lines were infected with OC43 coronavirus, RNA purified after 24 h, then relative viral load quantified via RT-qPCR with primers for OC43 genomic RNA (gRNA) and GAPDH. **[b]** Mixed populations of wild-type and vt1-TKO HEK293T cells were seeded, infected with OC43 coronavirus ("OC43 Inf. +") or mock infected ("OC43 Inf. -"), grown under standard conditions for 48 or 72 h, then harvested. The cell lines' proportions were assessed via gDNA isolation, PCR with indicated primer sets ("Primers"; see Fig. 2a), and agarose gel electrophoresis (top). The prevalence of vt1-TKO versus WT cells ("Gene Ratio") is the ratio of "ko" and "wt" product bands, each normalized to their respective "adj" product band. Quantifications of fraction knockout cells are shown as bar graphs (bottom).

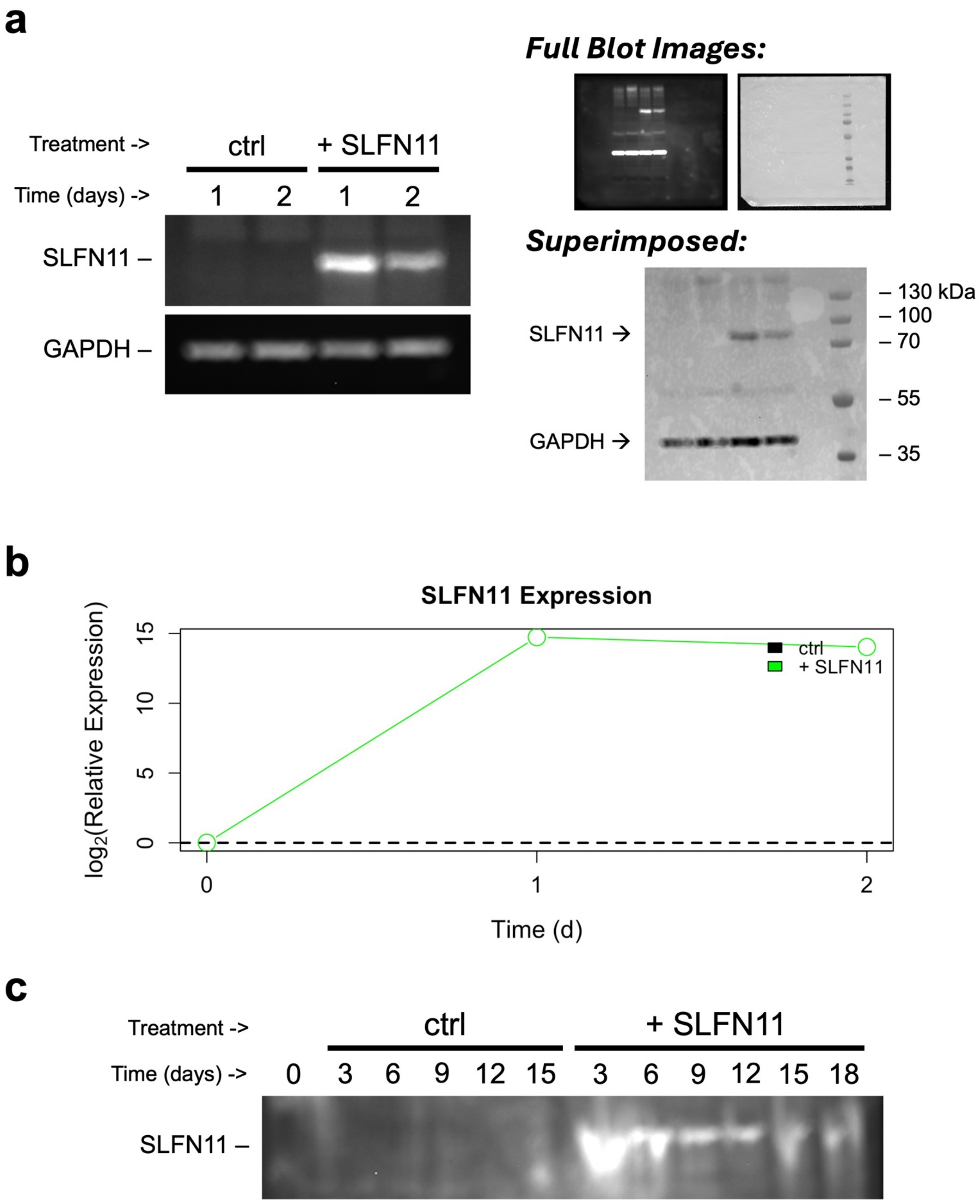

SUPPLEMENTAL FIGURE 7. SLFN11 is successfully overexpressed in HEK293T cells. [a] Wild-type HEK293T cells were grown under standard conditions ("ctrl") or with SLFN11 overexpression ("+ SLFN11"), sampled at 1 and 2 d post-transfection, and probed for SLFN11 expression by western blot with GAPDH loading control. SLFN11 and GAPDH bands shown (left images) are from two separate exposures of the same blot. Probe targets ("Full Blot Images", left) and protein standard ("Full Blot Images", right) were visualized with

separate channels, then the blot images superimposed for molecular weight comparison (“Superimposed”). Respective band identities are indicated at left of images, and sizes for protein standard are indicated at right. **[b]** Quantification of relative SLFN11 RNA expression from HEK293T cell samples in panel-a experiment. Values were first normalized to GAPDH, then to the control (“ctrl”, dashed black line) sample on the same day. Day-0 relative expression is inferred. **[c]** Western blot of SLFN11 protein expression in mixed HEK293T cell samples from Figure 5 experiments.

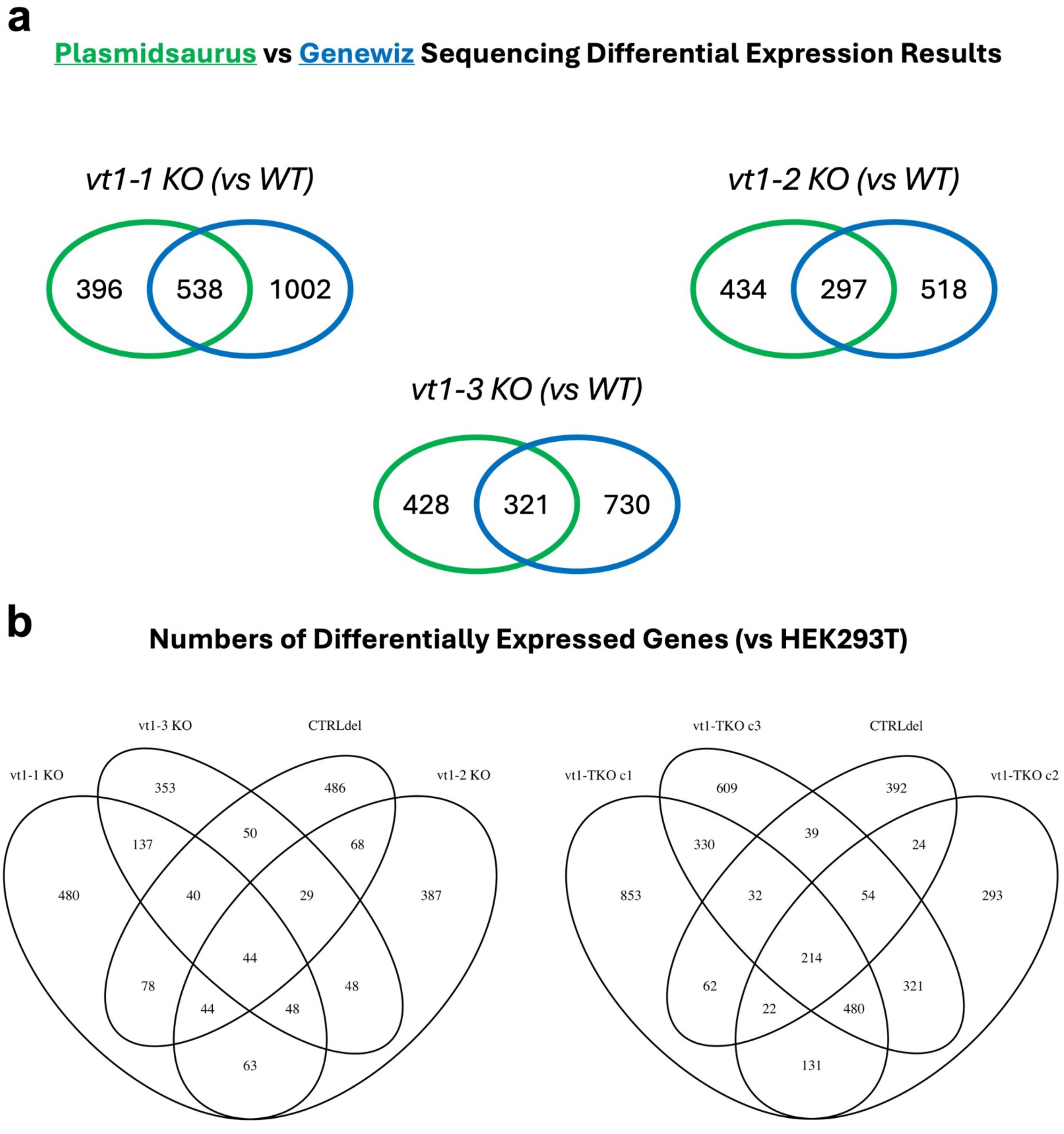

SUPPLEMENTAL FIGURE 8. Differential expression results are significantly influenced by clonal variation and sequencing approach. [a] Overlap between indicated RNA-sequencing methods in genes identified as differentially expressed in indicated knockout cell lines (versus wild-type; FDR < 0.05 & $\log_2|FC| > 1$). **[b]** Overlap between indicated knockout cell lines in genes identified as differentially expressed (versus wild-type; FDR < 0.05 & $\log_2|FC| > 1$) from the Plasmidsaurus sequencing set.

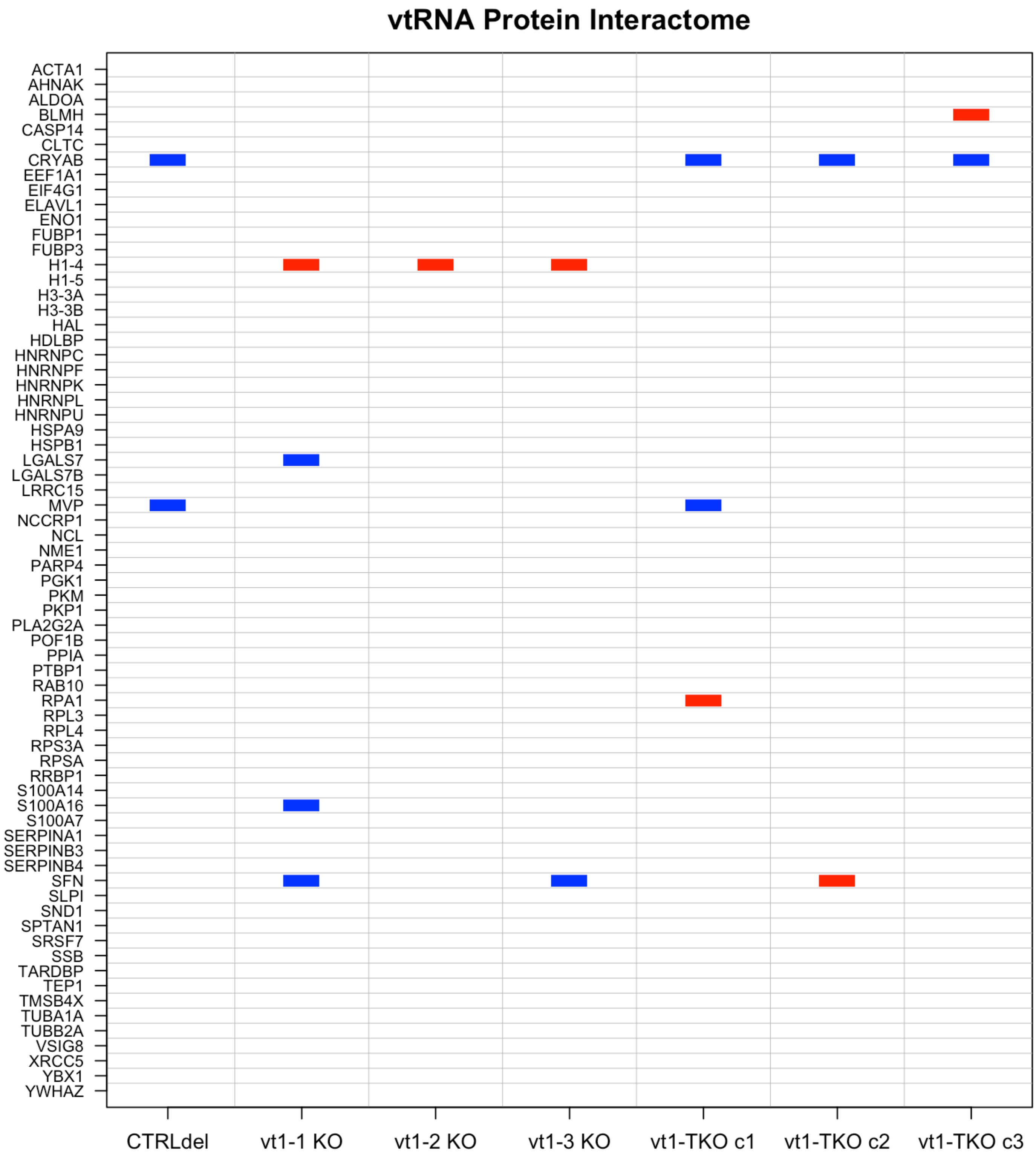

SUPPLEMENTAL FIGURE 9. Suspected vtRNA-binding proteins are differentially expressed in HEK293T cells lacking vtRNA1 paralogs. Suspected vtRNA-binding proteins implicated in (Stok et al. 2025) were identified in our differential expression data (Fig. 5b). Red and blue respectively correspond to down- and upregulation (FDR < 0.05 & $\log_2|FC| > 1$) of the indicated genes in the indicated cell lines.
